# Identification of A Novel Link between the Intermediate Filament Organizer IFO-1 and Cholesterol Metabolism in the *Caenorhabditis elegans* Intestine

**DOI:** 10.3390/ijms21218219

**Published:** 2020-11-03

**Authors:** Richard A. Coch, Florian Geisler, Andrea Annibal, Adam Antebi, Rudolf E. Leube

**Affiliations:** 1 Institute of Molecular and Cellular Anatomy, RWTH Aachen University, 52074 Aachen, Germany; rcoch@ukaachen.de (R.A.C.); fgeisler@ukaachen.de (F.G.); 2 Max Planck Institute for Biology of Ageing, 50931 Cologne, Germany; Andrea.Annibal@age.mpg.de (A.A.); antebi@age.mpg.de (A.A.); 3 Cologne Excellence Cluster on Cellular Stress Responses in Aging-Associated Diseases (CECAD), University of Cologne, 50931 Cologne, Germany

**Keywords:** intestine, *Caenorhabditis elegans*, endotube, cytoskeleton, intermediate filament, nuclear hormone receptor, lipid metabolism, cholesterol, fatty acid

## Abstract

The intestine is an organ essential to organismal nutrient absorption, metabolic control, barrier function and immunoprotection. The *Caenorhabditis elegans* intestine consists of 20 cells harboring a dense intermediate filament network positioned below the apical plasma membrane that forms a junction-anchored sheath around the intestinal lumen. This evolutionarily conserved arrangement provides mechanical and overall stress-protection, and it serves as an important model for deciphering the role of intestinal architecture in metazoan biology. We recently reported that the loss-of-function mutation of the intestinal intermediate filament organizer IFO-1 perturbs this architecture, leading to reduced body size and reproduction. Here, we demonstrate that the IFO-1 mutation dramatically affects cholesterol metabolism. Mutants showed an increased sensitivity to cholesterol depletion, reduced cholesterol uptake, and cholesterol transfer to the gonads, which is also observed in worms completely lacking an intermediate filament network. Accordingly, we found striking similarities to transcriptome and lipidome profiles of a nuclear hormone receptor (NHR)-8 mutant. NHR-8 is homologous to mammalian LXR (liver X receptor) that serves as a sterol sensor and transcriptional regulator of lipid metabolism. Remarkably, increasing exogenous cholesterol partially rescues the developmental retardation in IFO-1 mutants. Our results uncover a novel link of the intestinal intermediate filament cytoskeleton to cholesterol metabolism that contributes to compromised growth and reproduction.

## 1. Introduction

The intestinal epithelium delineates the border between the exterior environment and the body interior. It serves a dual function by providing a protective barrier against the harmful contents passing through the intestinal lumen and by facilitating the secure uptake of biomolecules needed for organismal metabolism. Elucidating these seemingly opposing properties and identifying the contributing cellular components are keys to understanding the physiologic function of the gut. The simplicity of the *Caenorhabditis elegans* intestine provides an ideal in vivo system to study intestinal structure–function relationships. The adult intestine consists of only 20 cells that are arranged as nine twisted rings (from int1 to int9), consisting of four (int1) or two cells (from int2 to int9) [1]. The polarized cells are tightly attached to each other by the *C. elegans* apical junction (CeAJ), which serves as an anchorage site for a dense periluminal lattice, the endotube that is composed, for the most part, of tightly packed intermediate filaments [2]. Of the six cytoplasmic, intermediate filament polypeptides in the *C. elegans* intestine, IFB-2 (intermediate filament polypeptide) serves as a master regulator, and the deletion of IFB-2 completely eliminates the endotube [3,4]. This property may be due to the abundance of IFB-2 and its capacity to polymerize with all other intestinal, intermediate filament polypeptides [5].

Genetic screens have identified various upstream regulators of intestinal network organization [6,7]. Among them is the intestinal intermediate filament organizer IFO-1 [6]. Its deletion induces a collapse of the entire intermediate filament network into large aggregates, most of which accumulate at the CeAJ. At the same time, apical F (filamentous)-actin is also reduced [6]. The loss-of-function *ifo-1(kc2)* mutants are small and have a reduced progeny production [6], indicative of a nutritional deficiency likely due to a nutrient uptake defect [6].

In the current study, we exploited the dependency of *C. elegans* on dietary cholesterol uptake to ask what contribution gut architecture plays in cholesterol homeostasis that it cannot synthesize on its own [8,9,10]. However, only minimal amounts (2.5–30 ng/mL of culture medium) are needed to sustain normal growth and reproduction [10,11]. In contrast to vertebrates, cholesterol is not an obligatory structural membrane component in *C. elegans*, although it is essential for steroid hormone biosynthesis [10,12]. In the complete absence of cholesterol, embryos obtained from parents that have been grown in the presence of cholesterol develop into adults (first filial generation; F1) and give rise to F2 progeny, albeit at reduced levels. The F2 embryos develop but completely arrest as dauer-like larvae with incomplete molting [13]. We found that *ifo-1(kc2)* mutants are sensitive to cholesterol deprivation, leading to complete larval arrest in the F1 generation. We further found that the uptake and transfer of cholesterol to progeny is impaired in *ifo-1(kc2)*. The impairment of cholesterol uptake and transfer was also observed in *ifb-2(kc14)* loss-of-function mutants, thus linking the intermediate filament cytoskeleton to dysfunction in cholesterol metabolism. A functional link between the IFO-1-controlled cytoskeleton and lipid metabolism was further supported by a significant overlap between the transcriptome and lipidome profiles of *ifo-1(kc2)* and *nhr-8(hd117)*. *nhr-8* encodes a nuclear hormone receptor homologous to mammalian LXR, which acts as a sterol sensor that regulates lipid and sterol metabolism [14]. These findings reveal a new and hitherto unknown contribution of IFO-1 and the intermediate filament cytoskeleton to organismal cholesterol and lipid metabolism.

## 2. Results

### 2.1. Lipids Are Reduced in the Intestine of ifo-1 Mutants

**Figure 1:**
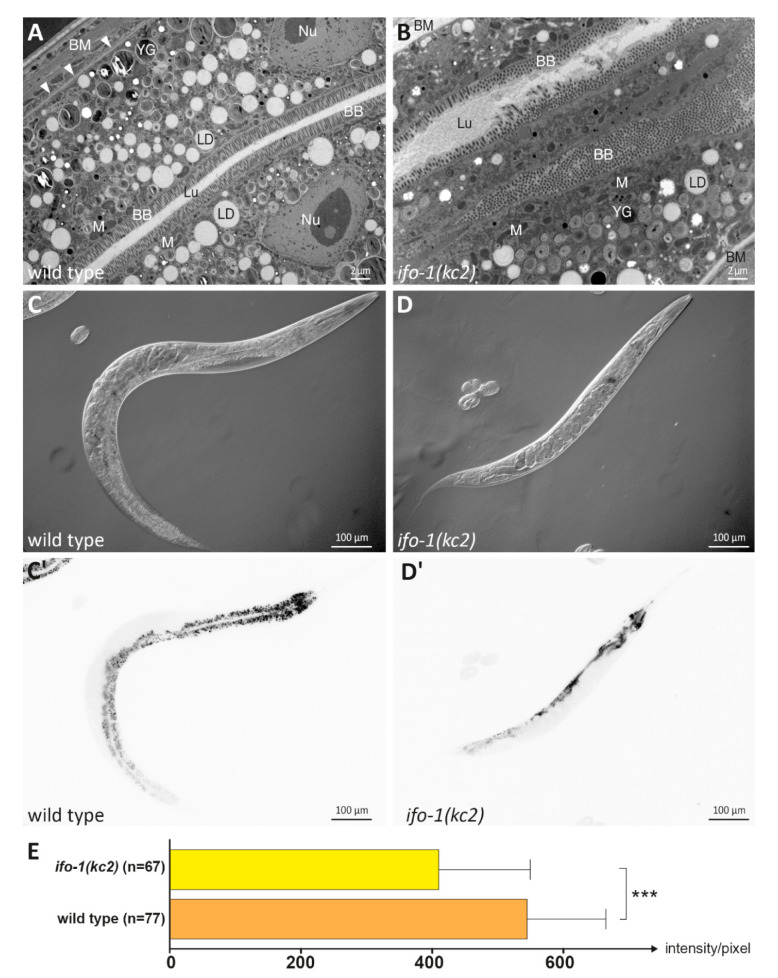
A slight reduction in lipid droplets and lipophilic Nile red stain was detected in the *ifo-1(kc2)* intestine of young adults. (**A** and **B**) The electron micrographs show a comparison of intestinal wild-type N2 and *ifo-1(kc2)* cytoplasm. Note the reduction of lipid droplets (LDs) in the mutant. YG: yolk granule; BB: brush border; Lu: intestinal lumen; BM: basement membrane (marked by arrowheads in **A**); M: mitochondrion; Nu: nucleus. (**C–D’**) The differential interference (**C** and **D**) and corresponding fluorescence images (**C’** and **D’**) present a comparison of Nile red staining in wild-type (**C** and **C’**) and *ifo-1(kc2)* worms (**D** and **D’**). (**E**) The histogram depicts the average fluorescence intensity in the intestinal cytoplasm of wild type (544.9 ± 119.8; *n* = 77) and *ifo-1(kc2)* (410.3 ± 139.4; *n* = 67) strains. Unpaired t-test; *** *p* < 0.001.

The loss-of-function allele *kc2* of the intestinal intermediate filament organizer gene *ifo-1* leads to reduced body size and progeny production [6], which may be caused by compromised food uptake in the intestine. Functional and biochemical evidence for this idea, however, is still lacking. Ultrastructural analyses of intestinal cells revealed luminal widening, disordered microvilli, and large intermediate filament polypeptide-containing aggregates at cell–cell junctions [6]. A close look at the cytoplasm of the mutant intestine suggested a reduction in the electron-lucent lipid droplets, while the amount of the darker yolk granules seemed not to be altered (Figure 1A,B). To quantify differences in lipid droplet composition, worms were stained with the lipophilic dye Nile red (for discussion of specificity, see [15]). A small but significant decrease in Nile red fluorescence was detected in *ifo-1(kc2)* compared to wild-type control animals (Figure 1C–E).

### 2.2. ifo-1 Mutants Exhibit Increased Sensitivity to Cholesterol Depletion

**Figure 2:**
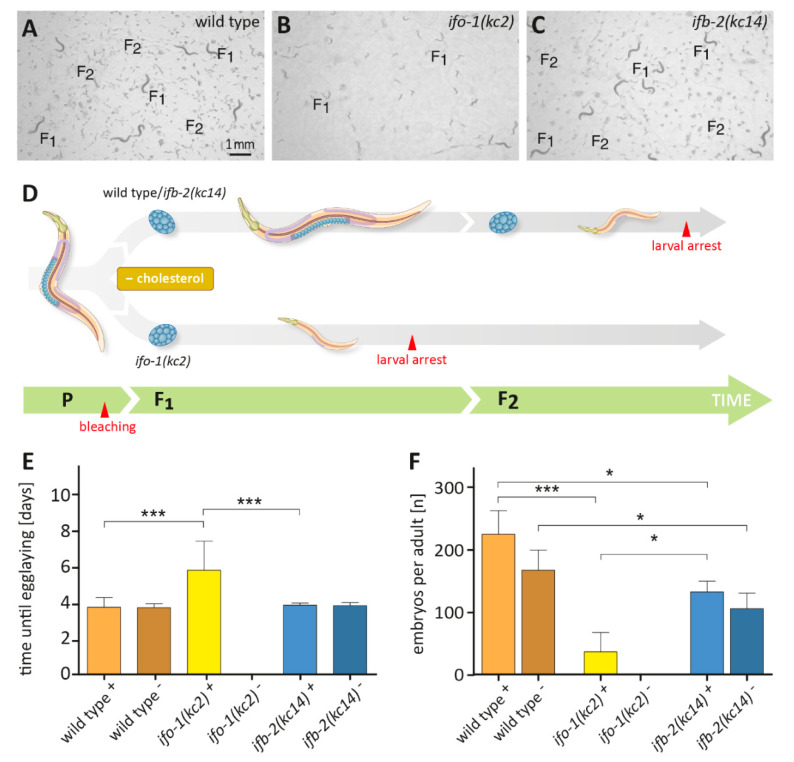
*ifo-1(kc2)* mutants arrest as larvae in F1 in the absence of cholesterol. (**A**–**D**) The pictures show representative images of cholesterol-free agarose plates at 4 days after the placement of wild-type N2 (**A**), *ifo-1(kc2)* (**B**), and *ifb-2(kc14)* embryos (**C**; same magnification in **A**–**C**), as well as a corresponding scheme of the different responses to cholesterol depletion. Note that in the absence of cholesterol, adult F1 animals are seen together with F2 embryos and larvae in both wild type and *ifb-2(kc14)*. In contrast, the F1 progeny of *ifo-1(kc2)* did not develop beyond early larval stages. (**E**) The histogram shows the time of development until egg laying in the presence (+) or absence (-) of cholesterol for wild-type N2 (+ cholesterol: 3.9 ± 0.6 days [*n* = 10]; - cholesterol: 3.9 ± 0.2 days [*n* = 9]), *ifo-1(kc2)* (+ cholesterol: 6.1 ± 1.7 days [*n* = 7]; - cholesterol: no development because of complete F1 larval arrest), and *ifb-2(kc14)* (+ cholesterol: 4.0 ± 0.1 days [*n* = 16]; - cholesterol: 4.0 ± 0.2 days [*n* = 16]). (**F**) The histogram depicts the progeny per animal in the presence (+) or absence of cholesterol (-) for wild-type N2 (+ cholesterol: 225 ± 37 [*n* = 11]; - cholesterol: 168 ± 32 [*n* = 9]), *ifo-1(kc2)* (+ cholesterol: 37 ± 31 [*n* = 7]; - cholesterol: none because of complete F1 larval arrest), and *ifb-2(kc14)* (+ cholesterol: 133 ± 17 [*n* = 15]; - cholesterol: 106 ± 25 [*n* = 16]). Kruskal–Wallis test: *** *p* < 0.001; * *p* < 0.05.

Because lipid metabolism is influenced by cholesterol [14], we next asked whether mutants showed an altered cholesterol homeostasis. Embryos were placed on cholesterol-depleted plates, and their development was followed. Wild-type N2 embryos developed into adults and reproduced in the absence of cholesterol. However, they had fewer progeny in the F2 generation, which arrested at the L1/L2 stage (for comparable observations, see [13]). In contrast, *ifo-1(kc2)* mutant embryos did not develop into adults in the absence of cholesterol but arrested as L1/L2 larvae already in the F1 generation (Figure 2A,B,D).

To find out whether the *ifo-1(kc2)*-dependent larval arrest phenotype was caused by the re-organization of the intermediate filament-containing endotube, we made use of *ifb-2(kc14)* knockout animals, which lack an endotube altogether [3,4]. Monitoring the growth of *ifb-2(kc14)* in the absence of cholesterol surprisingly showed that they produced F2 progeny, which, similar to the wild type, arrested at early larval stages (Figure 2C,D). Additional analyses revealed that the time of development of *ifb-2(kc14)* was not different from N2 (Figure 2E). Furthermore, the brood size was reduced in comparison to N2 (see also [3]), which, however, was independent of the presence or absence of cholesterol (Figure 2F). These findings suggested that the increased sensitivity of *ifo-1(kc2)* to cholesterol depletion may be caused by the junctional intermediate filament aggregates. On the other hand, the loss of intermediate filament polypeptides in *ifb-2(kc14)* did not affect cholesterol-dependency in the performed assay.

### 2.3. Cholesterol Uptake and Transport Is Reduced in ifo-1 and ifb-2 Mutants 

**Figure 3:**
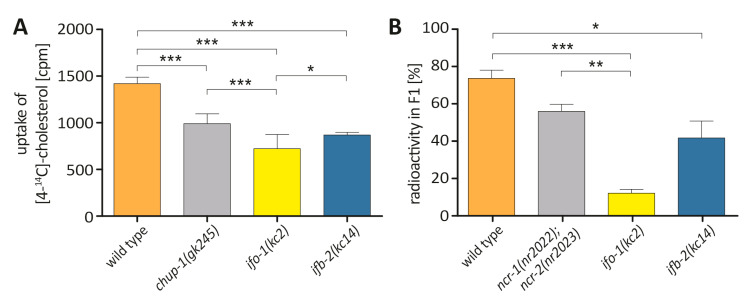
Cholesterol uptake and transfer is compromised in *ifo-1(kc2)* and *ifb-2(kc14)* mutants. (**A**) The histogram depicts the measured uptake of radioactively labeled [4-^14^C]- cholesterol into wild-type N2 (1419 ± 70 cpm; *n* = 8), *chup-1(gk245)* (991 ± 104 cpm; *n* = 8), *ifo-1(kc2)* (722 ± 152 cpm; *n* = 7), and *ifb-2(kc14)* (869 ± 28 cpm; *n* = 8). One-way ANOVA: *** *p* < 0.001; * *p* < 0.05. (**B**) The histogram shows the percentage of total incorporated radioactive cholesterol transferred to the F1 progeny for wild-type N2 (73.5 ± 4.4%; *n* = 5), *ncr-1(nr2022); ncr2(nr2023)* (55.8 ± 2.3%; *n* = 8), *ifo-1(kc2)* (12.0 ± 2.0%; *n* = 6), and *ifb-2(kc14)* (41.5 ± 9.0%; *n* = 8). Kruskal–Wallis test: *** *p* < 0.001; ** *p* < 0.01; * *p* < 0.05.

The above findings suggested that the uptake of cholesterol and/or cholesterol transport to the developing embryos are compromised in the *ifo-1(kc2)* mutants. To distinguish between them, worms were fed with radioactive cholesterol for measurements of cholesterol uptake and cholesterol transfer to the developing progeny. *chup-1(gk245)* was used as a control for the first set of experiments because it has been shown to impede cholesterol uptake [16]. The wild-type *chup-1* gene encodes an intestinal cholesterol transporter, the precise localization of which, however, has not been resolved to date [16]. Figure 3A illustrates that cholesterol uptake was significantly reduced in *chup-1(gk245)*. An even stronger reduction was detected in *ifo-1(kc2)*. A reduction in cholesterol uptake was also noted in *ifb-2(kc14*). Yet this reduction was less pronounced than in *ifo-1(kc2)* but similar to that in *chup-1(gk245).*

To quantify the transfer of cholesterol to progeny, cholesterol content was determined after radioactive cholesterol uptake in the F1 generation (Figure 3B). *ncr-1(nr2022)* and *ncr-2(nr2023)* double mutants were used in this instance as controls. *ncr-1* and *ncr-2* have been implicated in intracellular sterol transport [17]. A slight but statistically insignificant reduction was noted for the double mutant in comparison to the wild type. A significantly reduced cholesterol transfer, however, was detected for *ifb-2(kc14)* and was much more pronounced for *ifo-1(kc2)*.

Taken together, we conclude that *ifo-1* and *ifb-2* are both needed for cholesterol uptake and transport, albeit to different degrees. The lesser effect of the *ifb-2* mutation may explain why overall development is affected in *ifo-1(kc2)* but not in *ifb-2(kc14)* (as is the case for *chup-1(gk245)*; [16]).

### 2.4. Transcriptome Profiling Identifies Striking Similarities between ifo-1 and nhr-8 Mutants 

**Figure 4:**
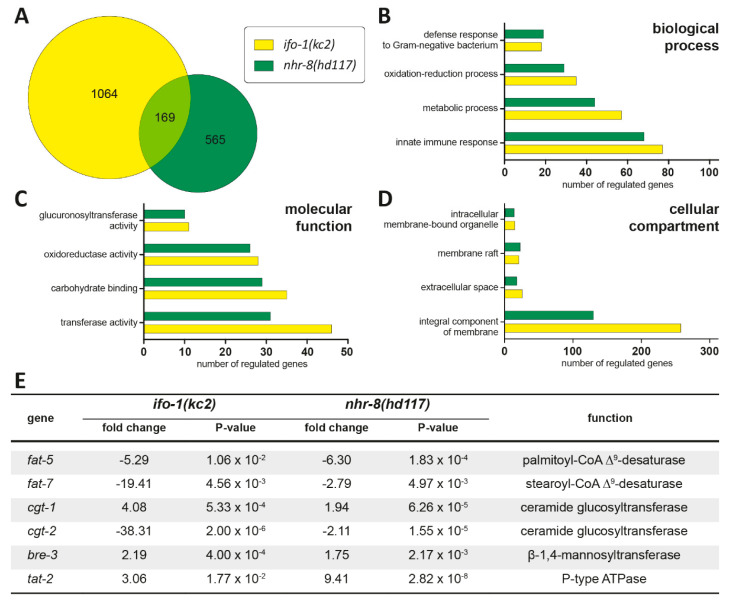
*ifo-1(kc2)* and *nhr-8(dh117)* share striking similarities in their transcriptomic profiles. (**A**) The Venn diagram shows the overlap (169 mRNAs) of differentially regulated mRNAs in *ifo-1(kc2)* (1064 mRNAs in dissected L4 intestines; yellow) and *nhr-8(hd117)* (565 mRNAs in the total lysates of L3 larvae growing at low cholesterol; green). The genes are listed in Table S1. (**B**–**D**) Results of gene ontology analysis using DAVID v6.8 are shown for biological process (**B**), molecular function (**C**), and cellular compartment (**D**). The numbers of differentially regulated genes in each category are depicted in yellow for *ifo-1(kc2)* and in green for *nhr-8(hd117)*. (**E**) The table lists genes that are relevant for lipid metabolism and are dysregulated in *ifo-1(kc2)* and *nhr-8(hd117)*.

Transcriptome analyses had been performed previously using dissected *ifo-1(kc2)* and wild-type intestines from L4 larvae to identify pathways that may be affected in the mutant [4]. When we compared the altered genes with other published mutants, considerable similarities were noted with the *nhr-8(hd117)* mutants [14]. *nhr-8* encodes a nuclear hormone receptor homologous to mammalian LXR and has been implicated as a sterol-sensor with important functions for nematode cholesterol homeostasis, lipid metabolism, and steroid hormone production [14]. Additionally, 169 of the dysregulated genes in *nhr-8(hd117)* total lysates that were obtained from L3 larvae grown under low cholesterol at 25 °C were also dysregulated in dissected *ifo-1(kc2)* intestines that were prepared from L4 larvae maintained at normal cholesterol at 20 °C (Figure 4A; Table S1). Only five of those genes were oppositely regulated in the different genetic backgrounds. No changes in the mRNA production for *ifo-1* in *nhr-8(hd117)* or, conversely, for *nhr-8* in *ifo-1(kc2)*, could be detected.

Gene ontology enrichment based on biological process, molecular function, and cellular compartment (Figure 4B–D) further underscored the significance of the overlapping gene expression profiles in *ifo-1(kc2)* and *nhr-8(hd117)*. Both mutant loci affected innate immune response, oxidative-reductive processes, and microbial defense, all of which have been implicated in the reduced stress resilience of *ifo-1* mutants [4] and appear to be co-regulated. Additionally, altered metabolic processes ranked second in the affected biological function of both mutant backgrounds. 

Among the differentially regulated genes, several stood out as important mediators of lipid metabolism (Figure 4E). These included *fat-5* and *fat-7*, which encode Δ9-desaturases (palmitoyl-CoA Δ9-desaturase and stearoyl-CoA Δ9-desaturase, respectively) that are involved in the synthesis of unsaturated fatty acids [18]. Moreover, the expression of *cgt-1*, *cgt-2*, and *bre-3*, which are part of the biosynthetic glycosphingolipid pathway, also showed dysregulation in both genetic backgrounds. Finally, *tat-2*, which encodes a P-type ATPase that has been implicated in sterol metabolism [19], was also altered in *ifo-1(kc2)* and *nhr-8(hd117)*.

### 2.5. Lipidomics Reveal Common Features of ifo-1, ifb-2 and nhr-8 Mutants 

**Figure 5:**
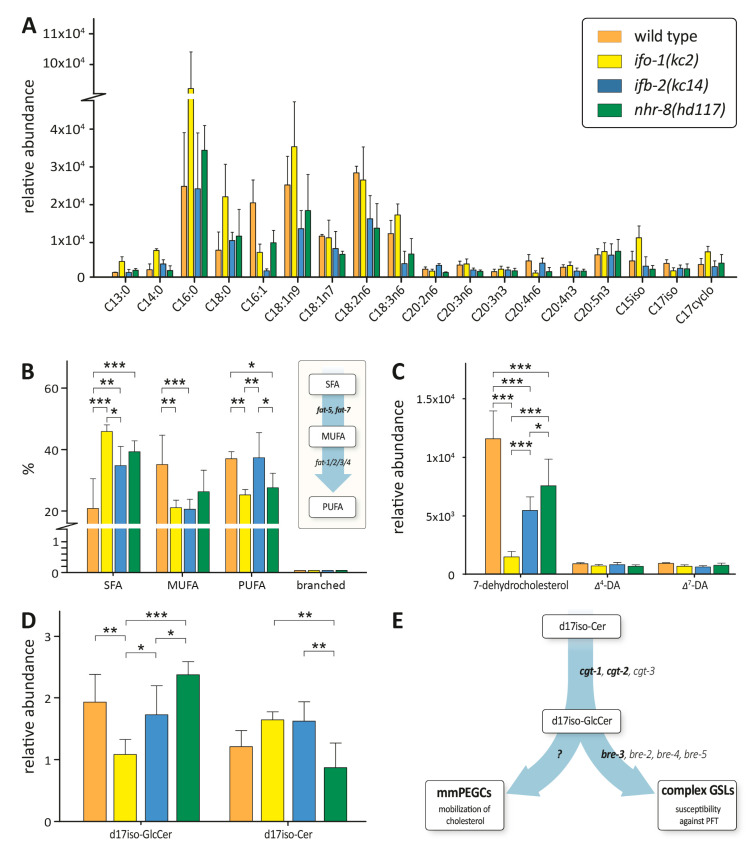
*ifo-1(kc2)*, *ifb-2(kc14)*, and *nhr-8(dh117)* share similarities in altered lipid profiles. (**A**–**D**) The histograms show the results of lipid analyses using gas chromatography–mass spectrometry (GC–MS), depicting the relative abundance (arbitrary units; *n* = 4) of all detected fatty acids (**A**); the percentage of saturated fatty acids (SFAs), monounsaturated fatty acids (MUFAs), polyunsaturated fatty acids (PUFAs), and branched fatty acids (branched; note that the amount is very low) (**B**); the relative abundance (arbitrary units) of the sterols 7-dehydrocholesterol as well as its derivatives Δ^4^- and Δ^7^-dafachronic acid (Δ^4^-DA and Δ^7^-DA, respectively) (**C**); and the glycosylated and non-glycosylated d17iso-containing ceramides (**D**) in N2, *ifo-1(kc2)*, *ifb-2(kc14)*, and *nhr-8(d117)*. The inset in (**B**) highlights the role of *fat-5* and *fat-7* for the production of MUFAs and PUFAs. Detailed results are provided in Table S2. Two-way ANOVA: *** *p* < 0.001; ** *p* < 0.01; * *p* < 0.05. (**E**) The scheme illustrates the influence of alterations in gene expression (regulated genes indicated in bold) and changes in the biosynthesis of sphingolipids potentially affecting the mobilization of cholesterol through the action of monomethylated phosphoethanolamine glucosylceramides (mmPEGCs; [22]) and susceptibility against pore-forming toxins (PFTs) mediated by complex glycosphingolipids (GSLs; [4,22,23]).

Based on the observed alterations in gene transcription, we asked whether lipid composition in *ifo-1(kc2)* and *ifb-2(kc14)* was affected and how they compared to the lipid composition in *nhr-8(hd117)*. A fatty acid methyl ester (FAME) analysis of fatty acid composition showed similar though not identical changes of saturated and unsaturated fatty acids in *ifo-1(kc2)* and *nhr-8(hd117)* (Figure 5A). A comparison of the percentage of saturated fatty acids (SFAs), monounsaturated fatty acids (MUFAs), polyunsaturated fatty acids (PUFAs), and branched fatty acids showed an increase in SFAs in all mutant backgrounds (Figure 5B). A significant decrease in MUFAs was observed in *ifo-1(kc2)* and *ifb-2(kc14)*, and only a minor decrease was observed in *nhr-8(hd117)*. This reduction was in line with the observed downregulation of *fat-5* and *fat-7* mRNA expression in *ifo-(kc2)* (Figure 4E). A comparable downregulation, together with a significant reduction in MUFAs, however, has been reported for *nhr-8(hd117)* grown at a low cholesterol level [14]. PUFAs were decreased in *ifo-1(kc2)* and in *nhr-8(hd117)* but not in *ifb-2(kc14)*. The role of PUFAs for lipid delivery to oocytes was recently shown by coherent anti-Stokes Raman scattering (CARS) microscopy in live *C. elegans* [20]. A low amount of branched fatty acids was observed but was unchanged in all genetic backgrounds.

Biosynthetic intermediates of the cholesterol-derived steroid hormone dafachronic acid [21] were also quantified. We observed a reduction in 7-dehydrocholesterol that was more pronounced in *ifo-1(kc2)* than in *ifb-2(kc14)* but not in Δ^4^- and Δ^7^-dafachronic acid (Figure 5C). The reduced amount of 7-dehydrocholesterol was in line with the previously reported downregulation of the Rieske oxygenase-encoding *daf-36* in *nhr-8(hd117)* [14]. This likely occurs through mechanisms independent of *daf-36* transcription, since we did not see any changes in *daf-36* mRNA levels in in *ifo-1(kc2)* or *ifb-2(kc14)* mutants.

For ceramides and glycosylated ceramide species, *ifo-1(kc2)* and *ifb-2(kc14)* animals showed somewhat discordant behavior relative to *nhr-8* (Figure 5D). Thus, *ifo-1(kc2)* and *ifb-2(kc14)* animals showed a decrease in glycosylated d17iso-containing ceramides and an increase in non-glycosylated d17iso-containing ceramides in comparison to *nhr-8(hd117)*. In addition, glycosylated d17iso-containing ceramides were reduced in *ifo-1(kc2)* compared to the wild type and *ifb-2(kc14)* strain. The further processing of glycosylceramides might be influenced by the observed upregulation of *bre-3* mRNA, which encodes a β-1,4-mannosyltransferase, leading to the phenotypic alterations illustrated in Figure 5E. Note that *ifo-1(kc2)* but none of the other strains showed significantly reduced levels of glycosylated ceramides when compared to the wild type strain, which might contribute to the observed developmental retardation of *ifo-1(kc2)* animals even under normal culture conditions.

### 2.6. nhr-8(RNAi) Mimics the Effect of Cholesterol Depletion in ifo-1(kc2) and ifb-2(kc14)

**Figure 6:**
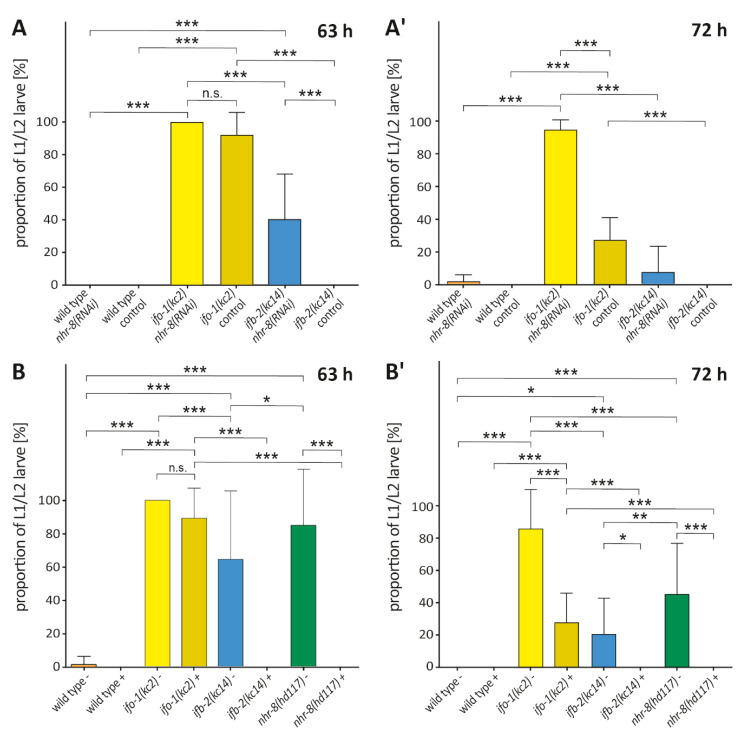
Interaction of *nhr-8* and cholesterol depletion with *ifo-1* and *ifb-2* reveals overlapping pathways. (**A** and **A’**) The diagrams show the percentages of early larval arrest after 63 h (**A**) for wild type *nhr-8(RNAi)* (0%; *n* = 12), wild type control (0%; *n* = 12), *ifo-1(kc2)/nhr-8(RNAi)* (100%; *n* = 12), *ifo-1(kc2)* control (92 ± 14%; *n* = 11), *ifb-2(kc14)/nhr-8(RNAi)* (40 ± 28%; *n* = 12), and *ifb-2(kc14)* control (0%; *n* = 12), as well as after 72 h (**A’**) for wild type *nhr-8(RNAi)* (1 ± 4%; *n* = 11), wild type control (0%; *n* = 12), *ifo-1(kc2)/nhr-8(RNAi)* (96 ± 6%; *n* = 12), *ifo-1(kc2)* control (28 ± 14%; *n* = 11), *ifb-2(kc14)/nhr-8(RNAi)* (7 ± 16%; *n* = 12), and *ifb-2(kc14)* control (0%; *n* = 12). (**B** and **B’**) The diagrams show the percentages of early larval arrest in the absence (-) or presence (+) of cholesterol after 63 h (**B**) for wild type - (1 ± 5%; *n* = 12), wild type + (0%; *n* = 12), *ifo-1(kc2)* - (100%; *n* = 12), *ifo-1(kc2)* + (89 ± 18%; *n* = 12), *ifb-2(kc14)* - (65 ± 41%; *n* = 11), *ifb-2(kc14)* + (0%; *n* = 12), *nhr-8(hd117)* - (85 ± 34%; *n* = 10), and *nhr-8(hd117)* + (0%; *n* = 11), as well as after 72 h (**B’**) for wild type - (0%; *n* = 12), wild type + (0%; *n* = 12), *ifo-1(kc2)* - (91 ± 17%; *n* = 12), *ifo-1(kc2)* + (28 ± 18%; *n* = 12), *ifb-2(kc14)* - (20 ± 22%; *n* = 11), *ifb-2(kc14)* + (0%; *n* = 12), *nhr-8(hd117)* - (45 ± 31%; *n* = 9), and *nhr-8(hd117)* + (0%; *n* = 12). Two-way ANOVA: *** *p* < 0.001; ** *p* < 0.01; *p < 0.05; n.s., not significant.

To examine the genetic interactions between *nhr-8* and *ifo-1/ifb-2*, *nhr-8* activity was downregulated by RNAi in *ifo-1(kc2)* and *ifb-2(kc14)* (Figure 6A,A’), with normal levels of cholesterol in the medium. We observed that after 63 h, *nhr-8(RNAi)* enhanced the weak *ifb-2(kc14)* phenotype but did not further enhance the already strong *ifo-1(kc2)* phenotype. Some worms were recovered from larval arrest after 72 h, presumably because animals were able to transport sufficient cholesterol through other pathways to grow with time or because of a subpar RNAi effect. The observations are consistent with the idea that *nhr-8* works in overlapping pathways with *ifo-1* and *ifb-2*.

We next asked whether these phenotypes could be modulated by cholesterol deprivation (Figure 6B,B’). As previously seen, *nhr-8(hd117)* animals halted development in early larval stages when grown in the absence of cholesterol [14]. Cholesterol depletion had no further effect on *ifo-1(kc2)* but enhanced *ifb-2(kc14)*, similar to *nhr-8(RNAi)* above. Notably, *ifo-1(kc2)* animals displayed L1/L2 arrest with and without cholesterol at 63 h (Figure 6B). At the later time point of 72 h, however, *ifo-1(kc2)* partly resumed development—more in the presence than in the absence of cholesterol (Figure 6B’). Residual traces of cholesterol in the used bacteria suspension may explain why some *ifo-1(kc2)* animals managed to escape larval arrest.

*ifb-2(kc14)* animals—which, in contrast, did not show any developmental defects under control conditions—displayed a comparable amount of larval arrest under both *nhr-8(RNAi)* and cholesterol deprivation, indicating a more subtle yet significant influence on *nhr-8*-regulated cholesterol handling that was not observed in the experiments shown in Figure 2E, possibly due to the use of normal growth medium (NGM) plates, instead of agarose plates, that were used for the experiments depicted in Figure 6. The obvious difference between *ifb-2(kc14)* and *ifo-1(kc2)* was most likely due to the more profound morphological changes and the presence of cytoplasmic and junctional intermediate filament polypeptide-containing aggregates in the *ifo-1(kc2)* background.

It should be noted that the comparable amount of larval arrest between *nhr-8(hd117)* - cholesterol and *ifo-1(kc2)* + cholesterol supports the hypothesis of reduced nuclear hormone receptor (NHR)-8 activity in *ifo-1(kc2)*. Our findings strongly suggest a regulatory role of the IFO-1-controlled intermediate filament network upstream of NHR-8.

### 2.7. High Cholesterol Concentration Partially Rescues ifo-1(kc2) Growth Retardation

**Figure 7:**
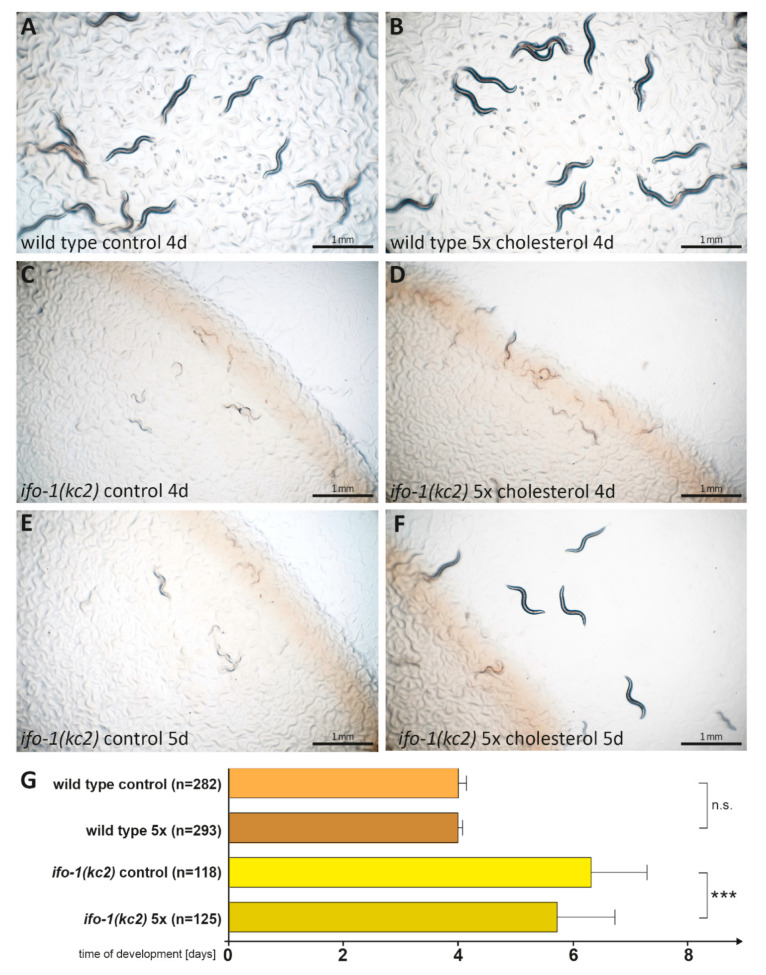
High cholesterol ameliorates the growth defect of *ifo-1(kc2)* animals. (**A**–**F**) Representative images show the developmental state of wild type (**A** and **B**) and *ifo-1(kc2)* animals (**C**–**F**) grown for 4 days (**A**–**D**) and 5 days (**E** and **F**) in control condition (**A**, **C**, and **E**) and in the presence of 5x cholesterol (**B**, **D**, and **F**). (**G**) The histogram depicts the time of development from embryo to adult for wild type and *ifo-1(kc2)* in control and 5× cholesterol (5×) conditions: wild type control (4.0 ± 0.1 days), wild type 5x (4.0 ± 0.1 days), *ifo-1(kc2)* control (6.3 ± 1.0 days), and *ifo-1(kc2)* 5x (5.7 ± 1.0 days). One-way ANOVA: *** *p* < 0.001; n.s. = not significant.

To address whether the slow development of *ifo-1(kc2)* animals is caused by a limited supply of exogenous cholesterol, we performed a rescue experiment with a medium containing five times the standard amount of cholesterol. Figure 7 shows that high cholesterol did not have any effect on the time of development of wild-type *C. elegans*, which complemented the observation that cholesterol deprivation also had no effect (Figure 2E). In contrast, the mean time of development was decreased in *ifo-1(kc2)* animals by 0.6 days in high cholesterol. This finding provides another piece of evidence for the cholesterol sensitivity of *ifo-1(kc2)*.

## 3. Discussion

**Figure 8:**
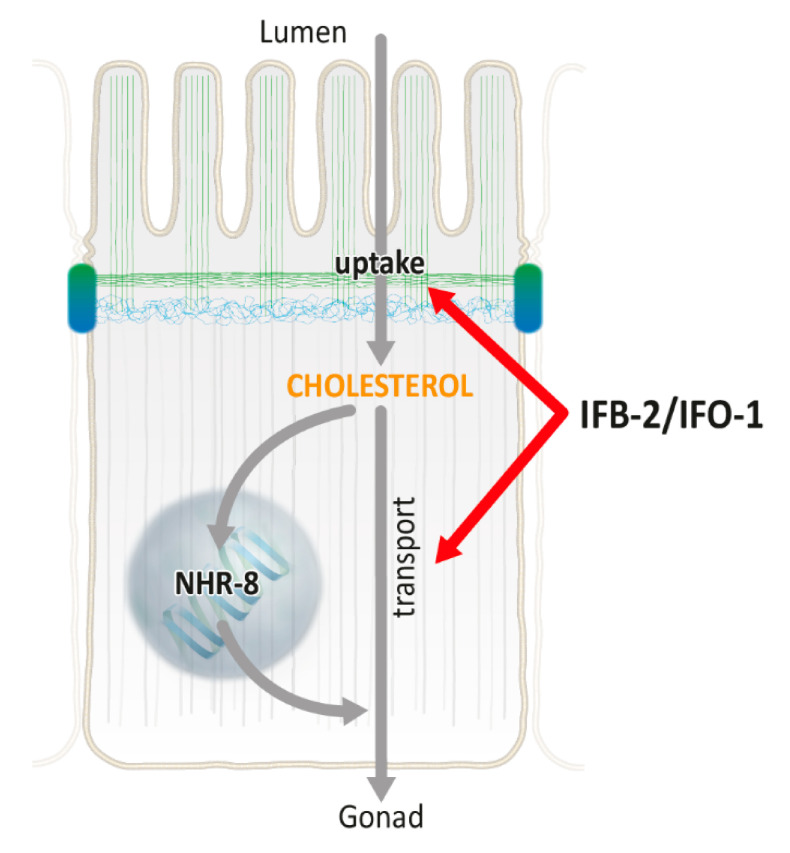
Schematic representation of possible interaction of IFO-1, IFB-2, and nuclear hormone receptor (NHR)-8 in the regulation of intestinal cholesterol transport. The uptake of cholesterol from the lumen, as well as the NHR-8-mediated transport of cholesterol-loaded yolk particles, depends on the apical intermediate filament cytoskeleton under control of IFO-1. Green: actin filaments; blue: intermediate filaments; and grey: microtubules. Top: apical microvillar brush border. Blue/green cell–cell contacts: *C. elegans* apical junction.

Our report identifies IFO-1 and the intermediate filament system as regulators of cholesterol and lipid metabolism. It adds to observations concerning mammalian vimentin, which is typically produced in non-epithelial mesenchymal cells and has been linked to lipid metabolism presumably by altering intracellular trafficking and organelle positioning [24,25,26]. In the present study, we observed an increased sensitivity to cholesterol depletion that was caused by reduced cholesterol uptake and transfer. Given the many open questions concerning intestinal cholesterol metabolism in vertebrates (review in [27,28]), and the limited observations in *C. elegans* [13,14,29], multiple scenarios can be envisioned (Figure 8). As a first step, cholesterol must be taken up from the intestinal lumen into the intestinal cell. In contrast to previous belief, it is not a simple, energy-independent diffusion process; instead, it is one that involves protein components such as Niemann-Pick Type C1-like protein 1 (NPC1L1), which is known to mediate cholesterol absorption [30]. Transport protein candidates in *C. elegans* are ChUP-1 [16] and NCR-1/NCR-2, which are related to NPC1L1 [17]. may the P-type ATPase TAT-2, whose mRNA is increased in *ifo-1(kc2)* (this study) and which has been implicated in sterol metabolism, may also be relevant [19]. Though the likely apical plasma membrane localization of these polypeptides remains to be shown, it is of interest to note that intermediate filaments contribute to the apical targeting of membrane proteins [31,32,33,34]. A simple hypothesis is that the loss of the apical intermediate filament network in *ifb-2(kc14)* and *ifo-1(kc2)* [3,6] leads to the inefficient localization of the above candidate transporters and/or other hitherto unidentified transporters. The observation that cholesterol transfer is less affected in *ncr-1(nr2022); ncr-2(nr2023)* in comparison to *ifo-1(kc2)* shows that the latter must involve other regulators of cholesterol metabolism. Similarly, the less pronounced inhibition of cholesterol uptake in *chup-1(gk245)* versus *ifo-1(kc2)* argues for the involvement of additional transporters. Furthermore, the more pronounced phenotype on cholesterol uptake of *ifo-1(kc2)* versus *ifb-2(kc14)* may be attributed to the accumulation of extensive intermediate filament-containing junctional aggregates that are uniquely seen in *ifo-1(kc2)* or to loss of IFO-1 functions that are independent of the intermediate filament system.

As a next step, cholesterol is transported through the cell. The scheme in Figure 8 highlights two aspects that are relevant to the present findings, i.e., the activation of the sterol sensor NHR-8 and additional mechanisms, that together lead to the formation of yolk granules that are released into the pseudocoelom for the nourishment of oocytes and developing embryos. We postulate that the reduced cholesterol uptake in *ifo-1(kc2)* and *ifb-2(kc14)* leads to the reduced activation of NHR-8. Several lines of evidence support this hypothesis.

(I) The overlap of transcriptional dysregulation between *nhr-8(hd117)* and *ifo-1(kc2)* (Figure 4) is quite remarkable considering that the compared microarray data stemmed from independent sources with different experimental setups. The analyses were done at different developmental stages (L3 versus L4), in different tissues (complete animals versus dissected intestines), and in different growth conditions (low versus normal cholesterol). This may account for the comparatively large number of differentially expressed, non-overlapping genes in the different genetic backgrounds (396 versus 895 genes). We cannot exclude that this also applies to some of the jointly regulated genes but would like to emphasize the striking similarities in concordant up- and down-regulated gene expression (164 of 169 genes).

(II) *ifo-1(kc2)* animals display striking phenotypic similarities with *nhr-8(hd117)* animals under low cholesterol conditions, notably a reduced uptake of cholesterol, an impaired supplementation of embryos with maternal cholesterol, and the accumulation of saturated fatty acids accompanied by a loss of mono- and poly-unsaturated fatty acids (Figure 3 and Figure 5; [14]).

(III) The knockdown of *nhr-8* by RNAi has the same consequence as cholesterol deprivation in *ifo-1(kc2)* animals, leading to early larval rest in the F1 generation (Figure 6).

The overlap in altered gene transcription of *ifo-1(kc2)*, which is predominantly localized in the apical cytoplasm [6], and *nhr-8(hd117)*, which localizes to both the cytoplasm and the nucleus [14], suggests that NHR-8 is a downstream target of pathways regulated by IFO-1. A consequence of this linkage are shared changes in lipid metabolism. Of interest is the reduction of PUFAs, which have been implicated in the delivery of lipids to the gonads [20]. The desaturases FAT-5 and FAT-7 may play a central role, since the transcription of the corresponding genes is considerably reduced in *ifo-1(kc2)* and *nhr-8(hd117)*. The resulting reduction in MUFAs is predicted to decrease production of PUFAs, which is catalyzed by FAT-1–4, as well as ELO-1 and ELO-2 [35]. Additionally of interest in this context is the reported reduction in overall intestinal lipid observed in *fat-6*/*fat-7* double mutants [18], which is in line with the reduced Nile red staining described in this report. Additionally, the knock down of the *C. elegans* sterol-regulatory-element-binding protein (SREBP) homolog SBP-1 also leads to the impaired expression of *fat-5* and *fat-7*, which is accompanied by growth defects, reduced fat content, and the accumulation of saturated fatty acids [36]. It remains to be shown whether low cholesterol availability in *ifo-1(kc2)*/*ifb-2(kc14)* affects SBP-1 activation.

The transcriptional dysregulation of the ceramide glucosyltransferases CGT-1 and CGT-2 is also shared by *ifo-1(kc2)* and *nhr-8(hd117)*. The much more pronounced downregulation of *cgt-2* in *ifo-1(kc2)* versus *nhr-8(hd117)* (Figure 4E) may explain the resulting decrease in d17iso-GlcCer in *ifo-1(kc2)* but not in *nhr-8(hd117)* (Figure 5D). The impaired synthesis of glycosphingolipids could be responsible for the growth defects in *ifo-1(kc2)* animals in accordance with previous reports, which have shown that the removal of CGT activity induces growth arrest [37,38]. In contrast to previous reports, which have ascribed important functions to CGT-1 and CGT-3, the present study additionally suggests the importance of CGT-2.

The upregulation of *bre-3* mRNA in *ifo-1(kc2)* and *nhr-8(hd117)* implicates another mechanism whereby lipid metabolism may be affected. The depletion of BRE-3 has been shown to increase intracellular cholesterol mobilization by increasing phosphoethanolamine glucosylceramides (PEGCs) [22]. Conceivably, the observed upregulation of *bre-3* mRNA might be responsible for the impaired mobilization of the intracellular cholesterol pool. 

The more severe phenotype of *ifo-1(kc2)* than that of *nhr-8(hd117)* and, conversely, the additive effect of *nhr-8(RNAi)* in *ifo-1(kc2)* suggest that NHR-8 is certainly not the only effector of IFO-1 and that, on the other hand, IFO-1-independent functions of NHR-8 exist. A major conundrum in this context is the downregulation of the *C. elegans* apolipoprotein genes *vit-1* and *vit-2* in *nhr-8(hd117)*, which is not observed in *ifo-1(kc2)* animals. Additionally, the transcription of the *daf-36* mRNA is reduced in *nhr-8(hd117)* but not in *ifo-1(kc2)*. *daf-36* encodes the Rieske oxygenase DAF-36, which catalyzes the reaction of cholesterol to 7-dehydrocholesterol. However, levels of 7-dehydrocholesterol are reduced not only in *nhr-8(hd117)* but also, and even more pronounced, in *ifo-1(kc2)*. A possible explanation for this paradox is the reduced availability of the DAF-36 substrate cholesterol in *ifo-1(kc2)* (Figure 3). It is unclear, however, why the levels of Δ^4^- and Δ^7^-dafachronic acids, which are derived from 7-dehydrocholesterol, are unchanged in *ifo-1(kc2)*, since *nhr-8* mutants show lower levels of these compounds under cholesterol depletion, which has been linked to dauer formation. It is possible that feedback mechanisms for the production of dafachronic acid can compensate [39,40].

Aside from their similarities, NHR-8 and IFO-1/IFB-2 also show differences. NHR-8-independent targets of IFO-1/IFB-2 may include components of the cytoplasmic lipid transport system. The mammalian vimentin intermediate filament cytoskeleton has been shown to interact with endosomal/lysosomal sorting machinery, thereby affecting the lysosomal exit of LDL (low-density lipoprotein)-derived cholesterol [41,42]. Moreover, the involvement of the NPC proteins in this process [43,44,45] raises the question of whether the localization or function of the *C. elegans* homologs NCR-1 and NCR-2 are affected in the *ifo-1/ifb-2* mutant backgrounds. 

Taken together, we conclude that the disruption of the apical intermediate filament network impairs the uptake of cholesterol, thus resulting in the dysregulation of cholesterol-dependent cellular processes and physiology that are, in part, mediated through NHR-8. A striking example of the physiological consequences of impaired cholesterol uptake was recently reported for mutants of *chup-1*, which was shown to be involved in the cholesterol- and NHR-8-dependent innate immune response to microbes [46]. The clarification of cholesterol-dependent molecular mechanisms connecting organismal growth, reproduction, and stress response with cytoskeletal organization, sterol sensing, and trafficking remains a great challenge. The cholesterol-dependence of *C. elegans* gut physiology offers an experimentally tractable scenario to address this. 

## 4. Material and Methods

### 4.1. Strains

Wild-type N2, the VC452 *chup-1(gk245)X* strain, the JT10800 *ncr-2(nr2023)III* strain, and the *ncr-1(nr2022)X* strain, as well as *Escherichia coli* strains OP50 and HT115, were obtained from the *Caenorhabditis* Genetics Center (CGC; University of Minnesota, USA). The BJ309 ifb-2(kc14)II, kcIs7[ifb-2p::ifb-2a[40-41insT]::cfp] *kcIs6]IV strain has been described in [3,4], and the BJ142 *ifo-1(kc2)IV* in [4,6] and strain AA968 *nhr-8(hd117)IV* strain was described in [14].

### 4.2. Nile Red Staining

The lipophilic dye Nile red, which becomes fluorescent in lipid-rich environments [47], was obtained from Fluka (Thermo Fisher Scientific Inc., Waltham, MA, USA). It was diluted to 0.5 ng/mL in water and was added to the bacterial lawn on agar plates followed by incubation overnight in the dark at room temperature. Plates were inoculated with L4 larvae and kept for 22 h at 20 °C. Worms were then transferred to an agarose pad and immobilized in 4 µL 1% levamisole (Sigma-Aldrich, St. Louis, MO, USA). Nile red fluorescence was assessed by microscopic imaging in a Zeiss Apotome at 10x magnification (Carl Zeiss AG, Oberkochen, Germany). Fluorescence was then quantified with the help of Fiji to manually define regions of interest and determine the grey values within.

### 4.3. Electron Microscopy

Electron microscopy was done on high-pressure frozen worms as described previously [3].

### 4.4. RNAi-Induced Larval Arrest

One mL of standard NGM agar containing 100 µg/mL ampicillin and 20 mM isopropyl β-d-1-thiogalactopyranoside (IPTG) was poured into each well of 24-well plates. The solidified agar was then inoculated with HT115 *E. coli* carrying either pPD129.36 empty vector or pPD129.36 containing *nhr-8* cDNA [48]. Worms were bleached (150 µL of 12% NaOCl, 100 µL of 4 M NaOH, and 1 mL of PBS) and washed with an M9 buffer (22 mM KH_2_PO_4_, 42 mM Na_2_HPO_4_, 86 mM NaCl, and 1 mM MgSO_4_), and then several of the expelled embryos were transferred into each well using a pulled-out capillary. Early larvae (L1/L2) and total offspring were counted after 63 and 72 h at 20 °C.

### 4.5. Cholesterol Depletion Assay

For cholesterol deprivation experiments, 10 cm agarose plates (1.75% (*w*/*v*) agarose, 52 mM NaCl, 25 mM KH_2_PO_4_/K_2_HPO_4_ [pH 6.0], 1 mM MgSO_4_, and 0.5 mM CaCl_2_) were prepared containing either 5 mg/L cholesterol in ethanol (control condition) or an equal amount of pure ethanol. The plates were inoculated with an overnight culture of OP50 *E. coli*, which was washed two times with the M9 buffer and re-suspended in 1/5 of its volume. Synchronized L1 larvae, which had hatched overnight on NGM plates without food and cholesterol, were transferred onto the test plates and incubated at 20 °C. To prevent overcrowding, a chunk of agar containing F2 larvae (if any) was transferred onto a fresh plate at day 3. Representative pictures were taken at days 4 and 6 using an SMZ1500 stereoscopic zoom microscope equipped with an µEye USB camera and a Plan Apo WD70 1× objective (Nikon Corporation, Tokyo, Japan).

For phenotypic analysis, 6 cm standard NGM plates with and without cholesterol were used. A single embryo of bleached animals was placed on each plate (see above) and incubated at 20 °C. The developmental stage was monitored daily, and gravid adults were transferred to a fresh plate each day to count the total offspring from each animal.

For the measurement of larval arrest, 1 mL of agarose (1.75% (*w*/*v*) agarose, 52 mM NaCl) with and without cholesterol, was poured into 24-well plates and inoculated with an overnight culture of OP50 *E. coli*. The experiment was started by placing several embryos of bleached animals into the wells (see above). Early larvae (L1/L2) and total offspring were counted after 63 and 72 h at 20 °C.

### 4.6. Cholesterol Uptake and Transfer Assays

Cholesterol feeding experiments were carried out at 20 °C on 10 cm agarose plates (1.75% (*w/v*) and 52 mM NaCl), inoculated with 800 µL of an overnight culture of OP50 *E. coli*, supplemented with 0.5 µCi of [4-^14^C]-cholesterol (Perkin-Elmer, Inc., Waltham, MA, USA). Synchronized L1 larvae, which had hatched overnight on NGM-plates without food and cholesterol, were placed on the feeding plates and allowed to grow to adulthood at 20 °C. The fed animals were washed two times with an M9 buffer, and groups of 20 animals each were either transferred to normal NGM plates or collected for lysis. The animals on the NGM plates were allowed to lay eggs for 3 days at 15 °C, after which they were removed and collected for lysis. After 2 days at 20 °C, the F1 generation was flushed off the plates, washed two times with an M9 buffer, and subjected to lysis. Lysis was carried out for 1 h at 65 °C in a single-worm lysis buffer, containing 0.5 µg/µL proteinase K. Afterwards, all samples were mixed with 5 mL of scintillation liquid Irgasafe plus (Perkin-Elmer, Inc., Waltham, MA, USA), and radioactivity was measured in a Hidex 600 SL liquid scintillation counter (Hidex Oy, Turku, Finland).

### 4.7. Cholesterol Rescue Experiment

Cholesterol rescue experiments were carried out on standard NGM-plates supplemented with either standard or 5× cholesterol. Worms were grown from synchronized embryos at 18 °C, and the developmental state was monitored daily.

### 4.8. Gene Ontology Analysis

Transcriptomic data for *nhr-8(hd117)* were previously published in [14], and transcriptomic data for *ifo-1(kc2)* were published in [4]. Both lists of regulated genes were subjected to gene ontology analysis using DAVID (database for annotation, visualization and integrated discovery) v6.8 using default settings [49].

### 4.9. Lipid Extraction and Analysis

The following materials were used for lipid extraction and analysis: UHPLC methanol, butylated hydroxytoluene, ammonium formate and ethyl acetate from Sigma-Aldrich (St. Louis, MO, USA); UHPLC grade water, UHPLC grade acetonitrile, and formic acid from Biosolve (Valkenswaard, The Netherlands); C4 ceramide (d18:1/4:0), C10 ceramide (d18:1/10:0), C12 GluCer (d18:1/12:0), and C17 Cer (d18:1/17:0) from Avanti Polar Lipids (Alabaster, AL, USA); isopropanol from Carl Roth (Karlsruhe, Germany); and chloroform from Merck (Darmstadt, Germany). In addition, the following standards were obtained: 7 dehydrocholesterol and 7 ketocholesterol (d7) were from Sigma-Aldrich (St. Louis, MO, USA), and Δ^7^-dafachronic acid and Δ^4^-dafachronic acid were from Biomol (Hamburg, Germany).

Lipid extraction from *C. elegans*: Worms were collected at young adult stage, and homogenates were obtained by beads beating for 30 min at 4 °C using a Qiagen TissueLyser (Qiagen, Venlo, The Netherlands); 0.1% (*w/v*) of butylated hydroxytoluene (BHT) was added to prevent autoxidation reactions. Protein concentrations were measured using the BCA assay kit (Thermo Fisher Scientific Inc., Waltham, MA, USA) prior to lipid analyses. A volume of homogenate corresponding to 600 µg of protein was used for ceramide analysis, a volume of homogenate corresponding to 200 µg of protein was used for fatty acid analysis (FAME), and a volume of homogenate corresponding to 200 µg of protein underwent solid phase extraction (SPE) for steroid enrichment (see corresponding sections).

Fatty acid analysis (FAME): GC–MS analyses were carried out on a GC/MS (Triple Quadrupole - GC 7890A + 7000 QQQ; Agilent Technologies, Santa Clara, CA, USA) interfaced with a robotic auto sampler PAL 1 system (PAL LHXxt). One microliter of the derivatized worm sample was injected into the liner (4 mm splitless, single taper liner with glass wool) by the pulsed-split mode at 200 °C. Analytes were separated using a DB Ultra Inert column (15, 250, and 0.25 mm; Agilent Technologies, Santa Clara, CA, USA). The initial oven temperature was set to 180 °C and held for 1 min, and then the temperature was increased by 30 °C/min up to 300 °C and held at that temperature for 1 min. At the end, the temperature was increased up to 325 °C by 30 °C/min. The temperature of the transfer line was set to 325 °C, and helium was used as the carrier gas at a flow rate of 1.4 mL/min in a constant flow mode. Ions were generated using an electron impact ion source (70 eV and 300°C) and analyzed by a triple quadrupole. C13:0 fatty acid was used as internal standard. Data were analyzed using MassHunter Workstation Software, Qualitative Analysis, Version B.06.00 (Agilent Technologies, Santa Clara, CA, USA). The relative response was calculated by dividing the peak area of the analyte to the internal standard peak area and further normalized to protein concentration.

Ceramide targeted analysis: Ceramides were extracted by adding a 2:1 (*v/v*) mixture of methanol and chloroform and incubated for 1 h at 4 °C. Samples were then centrifuged for 5 min at 3000 rcf at 4 °C, and the liquid phase was transferred and dried in a speed vac. Samples were reconstituted with 60:40 (*v/v*) methanol:water, and 5 µL were injected into the LC–MS system. The identification of ceramides and glucosylated ceramides was performed on a Q Exactive Mass Spectrometer (Thermo Fisher Scientific Inc., Waltham, MA, USA) coupled with a binary pump system (Vanquish, Thermo Fisher Scientific Inc., Waltham, MA, USA). Lipid species were separated using an RP column (Xbridge BEH C8 2.5 µm, 2.1 × 100 mm) using solvents A (60:40 *v/v* acetonitrile: water with 10 mmol/L ammonium formate) and B (90:10 *v/v* isopropanol:water with 10 mmol/L ammonium formate), as previously reported [50]. The gradient started from 32% B then ramped to 45% B in 4 min. In 1 min, the gradient was ramped till 52% B, and in 3 min, it was ramped till 58% B. Then, in 3 min, it increased up to 66% B before being ramped in 3 min till 70% B. In the following 4 min, it went till 75% B, and then it ramped in 3 min to 97% B and held for 4 min; finally, in 1 min, it went back to 32% B and held for 4 min. The total time was 30 min, and the column was heated at 45 °C using a flow rate of 100 µL/min. The LC system was flushed in between runs with 75/25 (*v/v*) isopropanol water with 0.1% formic acid.

Ceramides and glycosylated ceramides were detected in negative ion mode using an H-ESI ion source with the following parameters: sheath gas of 10, auxiliary gas of 2, spray voltage of 3 kV, and ion transfer tube temperature of 250 °C. Data were acquired using a targeted-selected ion monitoring chromatogram mode (t-SIM) with a resolution of 70.000, AGC 5 e^4^, a maximum injection time of 200 ms, and an isolation of 1.0 *m/z*. C4 Ceramide (d18:1/4:0) and C12 GluCer d18:1/12:0 were used as internal standards. Data were analyzed using Xcalibur version 4.0 and Trace Finder version 4.1 (Thermo Fisher Scientific Inc., Waltham, MA, USA). The relative response for each steroid species was calculated by dividing the peak area of the analyte to the internal standard peak area, and the result was further normalized to protein concentration.

Steroid analysis: HLB prime cartridges (Oasis, Waters, Eschborn, Germany) were pre conditioned with 1 mL of methanol and 1 mL of water with 1% (*v/v*) formic acid before adding the sample. The homogenate was directly applied to the column, and phospholipids were removed by adding 1 mL of hexane. Steroids were eluted using 500 µL of ethyl acetate, as previously described [51]. The organic fraction was dried in a speed vac. All samples were reconstituted in 20 µL of 50% (*v/v*) methanol/water, and 5 µL were injected into the LC–MS/MS system.

LC–MS analysis was carried out on a triple quadrupole mass spectrometer (QqQMS) (TSQ Altis; Thermo Fisher Scientific Inc., Waltham, MA, USA). Prior to the measurements, gradient optimization was performed using authentic standards. Steroids were separated with a reverse phase column (XSelect HSS T3 2.5 μm 2.1 × 100; Waters, Eschborn, Germany) using a binary pump system (Vanquish, Thermo Fisher Scientific Inc., Waltham, MA, USA) with solvent A as water with 0.1% (*v/v*) formic acid and eluent B as acetonitrile with 0.1% (*v/v*) formic acid; 2 µL of the reconstituted sample were injected. The flow rate was 0.1 mL/min for the first 5 min and then increased to 0.150 mL/min thereafter. The column temperature was set at 30 °C. The ESI ionization parameters were as follows: 3.5 kV, 25 a.u. sheath gas, 5 a.u. auxiliary, and 350 °C transfer ion capillary. All the spectra were acquired in positive ion mode.

For the internal standard, 7 ketocholesterol (d7) was used. Data were analyzed using Xcalibur version 4.0 and Trace Finder version 4.1. The relative response for each steroid species was calculated by dividing the peak area of the analyte to the internal standard peak area, and the result was further normalized to protein concentration.

## Supplementary Materials

Supplementary materials can be found at https://www.mdpi.com/1422-0067/21/21/8219/s1. Table S1: List of genes that are altered in both *ifo-1(kc2)* and *nhr8(hd117);* Table S2: List of lipid measurements in wild-type N2, *ifo-1(kc2)*, *ifb-2(kc14)* and *nhr-8(hd117).*

## References

[1] McGhee J.D. (2007). The *C. elegans* intestine. WormBook.

[2] Carberry K., Wiesenfahrt T., Windoffer R., Bossinger O., Leube R.E. (2009). Intermediate filaments in Caenorhabditis elegans. Cell Motil. Cytoskelet..

[3] Geisler F., Coch R.A., Richardson C., Goldberg M., Bevilacqua C., Prevedel R., Leube R.E. (2020). Intestinal intermediate filament polypeptides in C. elegans: Common and isotype-specific contributions to intestinal ultrastructure and function. Sci. Rep..

[4] Geisler F., Coch R.A., Richardson C., Goldberg M., Denecke B., Bossinger O., Leube R.E. (2019). The intestinal intermediate filament network responds to and protects against microbial insults and toxins. Development.

[5] Karabinos A., Schunemann J., Parry D.A. (2017). Assembly studies of six intestinal intermediate filament (IF) proteins B2, C1, C2, D1, D2, and E1 in the nematode *C. elegans*. Cytoskeleton.

[6] Carberry K., Wiesenfahrt T., Geisler F., Stocker S., Gerhardus H., Uberbach D., Davis W., Jorgensen E., Leube R.E., Bossinger O. (2012). The novel intestinal filament organizer IFO-1 contributes to epithelial integrity in concert with ERM-1 and DLG-1. Development.

[7] Geisler F., Gerhardus H., Carberry K., Davis W., Jorgensen E., Richardson C., Bossinger O., Leube R.E. (2016). A novel function for the MAP kinase SMA-5 in intestinal tube stability. Mol. Biol. Cell.

[8] Chitwood D.J. (1999). Biochemistry and function of nematode steroids. Crit. Rev. Biochem. Mol. Biol..

[9] Hieb W.F., Rothstein M. (1968). Sterol requirement for reproduction of a free-living nematode. Science.

[10] Kurzchalia T.V., Ward S. (2003). Why do worms need cholesterol?. Nat. Cell Biol..

[11] Merris M., Wadsworth W.G., Khamrai U., Bittman R., Chitwood D.J., Lenard J. (2003). Sterol effects and sites of sterol accumulation in Caenorhabditis elegans: Developmental requirement for 4alpha-methyl sterols. J. Lipid. Res..

[12] Watts J.L., Ristow M. (2017). Lipid and Carbohydrate Metabolism in Caenorhabditis elegans. Genetics.

[13] Matyash V., Entchev E.V., Mende F., Wilsch-Brauninger M., Thiele C., Schmidt A.W., Knolker H.J., Ward S., Kurzchalia T.V. (2004). Sterol-derived hormone(s) controls entry into diapause in Caenorhabditis elegans by consecutive activation of DAF-12 and DAF-16. PLoS Biol..

[14] Magner D.B., Wollam J., Shen Y., Hoppe C., Li D., Latza C., Rottiers V., Hutter H., Antebi A. (2013). The NHR-8 nuclear receptor regulates cholesterol and bile acid homeostasis in C. elegans. Cell Metab..

[15] Lemieux G.A., Ashrafi K. (2015). Insights and challenges in using C. elegans for investigation of fat metabolism. Crit. Rev. Biochem. Mol. Biol..

[16] Valdes V.J., Athie A., Salinas L.S., Navarro R.E., Vaca L. (2012). CUP-1 is a novel protein involved in dietary cholesterol uptake in Caenorhabditis elegans. PLoS ONE.

[17] Li J., Brown G., Ailion M., Lee S., Thomas J.H. (2004). NCR-1 and NCR-2, the C. elegans homologs of the human Niemann-Pick type C1 disease protein, function upstream of DAF-9 in the dauer formation pathways. Development.

[18] Brock T.J., Browse J., Watts J.L. (2007). Fatty acid desaturation and the regulation of adiposity in Caenorhabditis elegans. Genetics.

[19] Lyssenko N.N., Miteva Y., Gilroy S., Hanna-Rose W., Schlegel R.A. (2008). An unexpectedly high degree of specialization and a widespread involvement in sterol metabolism among the C. elegans putative aminophospholipid translocases. BMC Dev. Biol..

[20] Chen W.W., Yi Y.H., Chien C.H., Hsiung K.C., Ma T.H., Lin Y.C., Lo S.J., Chang T.C. (2016). Specific polyunsaturated fatty acids modulate lipid delivery and oocyte development in C. elegans revealed by molecular-selective label-free imaging. Sci. Rep..

[21] Aguilaniu H., Fabrizio P., Witting M. (2016). The Role of Dafachronic Acid Signaling in Development and Longevity in Caenorhabditis elegans: Digging Deeper Using Cutting-Edge Analytical Chemistry. Front Endocrinol..

[22] Boland S., Schmidt U., Zagoriy V., Sampaio J.L., Fritsche R.F., Czerwonka R., Lubken T., Reimann J., Penkov S., Knolker H.J. (2017). Phosphorylated glycosphingolipids essential for cholesterol mobilization in Caenorhabditis elegans. Nat. Chem. Biol..

[23] Griffitts J.S., Haslam S.M., Yang T., Garczynski S.F., Mulloy B., Morris H., Cremer P.S., Dell A., Adang M.J., Aroian R.V. (2005). Glycolipids as receptors for Bacillus thuringiensis crystal toxin. Science.

[24] Schweitzer S.C., Evans R.M. (1998). Vimentin and lipid metabolism. Subcell. Biochem..

[25] Evans R.M. (1994). Intermediate filaments and lipoprotein cholesterol. Trends Cell Biol..

[26] Shen W.J., Zaidi S.K., Patel S., Cortez Y., Ueno M., Azhar R., Azhar S., Kraemer F.B. (2012). Ablation of vimentin results in defective steroidogenesis. Endocrinology.

[27] Ko C.W., Qu J., Black D.D., Tso P. (2020). Regulation of intestinal lipid metabolism: Current concepts and relevance to disease. Nat. Rev. Gastroenterol. Hepatol..

[28] Wang D.Q. (2003). New concepts of mechanisms of intestinal cholesterol absorption. Ann Hepatol.

[29] Matyash V., Geier C., Henske A., Mukherjee S., Hirsh D., Thiele C., Grant B., Maxfield F.R., Kurzchalia T.V. (2001). Distribution and transport of cholesterol in Caenorhabditis elegans. Mol. Biol. Cell.

[30] Altmann S.W., Davis H.R., Zhu L.J., Yao X., Hoos L.M., Tetzloff G., Iyer S.P., Maguire M., Golovko A., Zeng M. (2004). Niemann-Pick C1 Like 1 protein is critical for intestinal cholesterol absorption. Science.

[31] Asghar M.N., Priyamvada S., Nystrom J.H., Anbazhagan A.N., Dudeja P.K., Toivola D.M. (2016). Keratin 8 knockdown leads to loss of the chloride transporter DRA in the colon. Am. J. Physiol. Gastrointest Liver. Physiol..

[32] Hou X., Wu Q., Rajagopalan C., Zhang C., Bouhamdan M., Wei H., Chen X., Zaman K., Li C., Sun X. (2019). CK19 stabilizes CFTR at the cell surface by limiting its endocytic pathway degradation. FASEB J..

[33] Toivola D.M., Krishnan S., Binder H.J., Singh S.K., Omary M.B. (2004). Keratins modulate colonocyte electrolyte transport via protein mistargeting. J. Cell Biol..

[34] Duan Y., Sun Y., Zhang F., Zhang W.K., Wang D., Wang Y., Cao X., Hu W., Xie C., Cuppoletti J. (2012). Keratin K18 increases cystic fibrosis transmembrane conductance regulator (CFTR) surface expression by binding to its C-terminal hydrophobic patch. J. Biol. Chem..

[35] Watts J.L., Browse J. (2002). Genetic dissection of polyunsaturated fatty acid synthesis in Caenorhabditis elegans. Proc. Natl. Acad. Sci. USA.

[36] Watts J.L. (2009). Fat synthesis and adiposity regulation in Caenorhabditis elegans. Trends Endocrinol Metab.

[37] Marza E., Simonsen K.T., Faergeman N.J., Lesa G.M. (2009). Expression of ceramide glucosyltransferases, which are essential for glycosphingolipid synthesis, is only required in a small subset of C. elegans cells. J. Cell Sci..

[38] Nomura K.H., Murata D., Hayashi Y., Dejima K., Mizuguchi S., Kage-Nakadai E., Gengyo-Ando K., Mitani S., Hirabayashi Y., Ito M. (2011). Ceramide glucosyltransferase of the nematode Caenorhabditis elegans is involved in oocyte formation and in early embryonic cell division. Glycobiology.

[39] Gerisch B., Antebi A. (2004). Hormonal signals produced by DAF-9/cytochrome P450 regulate C. elegans dauer diapause in response to environmental cues. Development.

[40] Motola D.L., Cummins C.L., Rottiers V., Sharma K.K., Li T., Li Y., Suino-Powell K., Xu H.E., Auchus R.J., Antebi A. (2006). Identification of ligands for DAF-12 that govern dauer formation and reproduction in *C. elegans*. Cell.

[41] Sarria A.J., Panini S.R., Evans R.M. (1992). A functional role for vimentin intermediate filaments in the metabolism of lipoprotein-derived cholesterol in human SW-13 cells. J. Biol. Chem..

[42] Styers M.L., Salazar G., Love R., Peden A.A., Kowalczyk A.P., Faundez V. (2004). The endo-lysosomal sorting machinery interacts with the intermediate filament cytoskeleton. Mol. Biol. Cell.

[43] Peake K.B., Vance J.E. (2010). Defective cholesterol trafficking in Niemann-Pick C-deficient cells. FEBS Lett..

[44] Vanier M.T. (2010). Niemann-Pick disease type C. Orphanet J. Rare Dis..

[45] Vanier M.T. (2015). Complex lipid trafficking in Niemann-Pick disease type C. J. Inherit. Metab. Dis..

[46] Otarigho B., Aballay A. (2020). Cholesterol Regulates Innate Immunity via Nuclear Hormone Receptor NHR-8. iScience.

[47] Greenspan P., Mayer E.P., Fowler S.D. (1985). Nile red: A selective fluorescent stain for intracellular lipid droplets. J. Cell Biol..

[48] Timmons L., Fire A. (1998). Specific interference by ingested dsRNA. Nature.

[49] Huang da W., Sherman B.T., Lempicki R.A. (2009). Systematic and integrative analysis of large gene lists using DAVID bioinformatics resources. Nat. Protoc..

[50] Tharyan R.G., Annibal A., Schiffer I., Laboy R., Atanassov I., Weber A.L., Gerisch B., Antebi A. (2020). NFYB-1 regulates mitochondrial function and longevity via lysosomal prosaposin. Nat. Metab..

[51] Dabrowski R., Ripa R., Latza C., Annibal A., Antebi A. (2020). Optimization of mass spectrometry settings for steroidomic analysis in young and old killifish. Anal. Bioanal. Chem..

